# Pregnancy, asthma and exacerbations: a population-based cohort

**DOI:** 10.1183/13993003.01327-2025

**Published:** 2025-12-18

**Authors:** Bohee Lee, Ernie Wong, Tricia Tan, Hitasha Rupani, Chloe I. Bloom

**Affiliations:** 1 National Heart and Lung Institute, Imperial College London, London, UK; 2 Department of Metabolism, Digestion and Reproduction, Imperial College London, London, UK; 3 Department of Respiratory Medicine, University Hospitals Southampton NHS Foundation Trust, Southampton, UK

## Abstract

**Background:**

Asthma exacerbations during pregnancy are associated with adverse maternal and perinatal outcomes. Identifying modifiable risk factors is essential for improving health outcomes. We aimed to describe exacerbation patterns during pregnancy and identify exacerbation risk factors, particularly modifiable risk factors such as inhaled corticosteroid use.

**Methods:**

This was a cohort study using UK primary care and hospital data (2004–2020) to identify pregnant women with asthma. Exacerbations were defined as a short course of oral corticosteroids, emergency department visit or unscheduled hospital admission. Multivariable logistic regression was used to assess associations between maternal characteristics and exacerbations (primary outcome) and inhaled corticosteroid use (secondary outcome).

**Results:**

Among 40 196 pregnant women with asthma, total exacerbations declined by ∼30% during pregnancy. However, exacerbations associated with hospital admission increased by 30–45% during the second and third trimesters, declining abruptly after delivery. Inhaled corticosteroid prescriptions were reduced in 31% of women during pregnancy. Decreased inhaled corticosteroid use was associated with suboptimal asthma control pre-pregnancy, age, ethnicity and smoking. The strongest exacerbation risk factors were a history of exacerbations (adjusted OR 4.09, 95% CI 3.81–4.39), reduced inhaled corticosteroid use during pregnancy (adjusted OR 2.29, 95% CI 2.12–2.47) and ≥4 prescriptions per year for inhaled corticosteroids plus another preventer before pregnancy (adjusted odds ratio 2.11, 95% CI 1.87–2.37). Additional risk factors included blood eosinophilia, smoking and obesity.

**Conclusions:**

Despite fewer total exacerbations, exacerbations associated with a hospital admission increased during pregnancy. One third of women reduced inhaled corticosteroid use during pregnancy, yet this was the second largest exacerbation risk factor and is completely modifiable. Other major risk factors were type 2 inflammation and another modifiable risk factor, suboptimal asthma control pre-pregnancy.

## Shareable abstract

**In a large pregnancy asthma cohort, hospitalised exacerbations increased. Many risk factors were modifiable. A third of women reduced their inhaled steroid prescriptions during pregnancy, doubling exacerbation risk. Other factors included suboptimal control and type 2 inflammation.** https://bit.ly/3Jri7OL

## Graphical abstract

**Figure:**
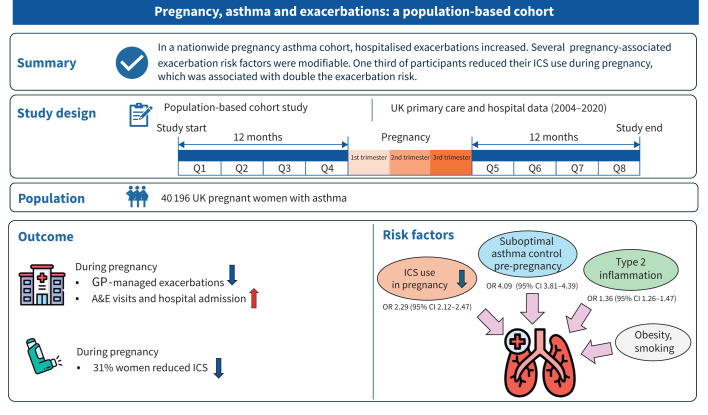
Overview of the study. A&E: accident and emergency department; GP: general practitioner; ICS: inhaled corticosteroids.

## Introduction

Asthma is the most common pre-existing chronic condition in pregnancy and asthma symptoms are known to manifest variably, including during pregnancy [1]. The Global Initiative for Asthma (GINA) notes that during pregnancy, “in approximately one third of women asthma symptoms worsen, in one third they improve, and in the remaining one third they remain unchanged” [2]. However, this commonly used adage is based on a single US cohort of 330 women from the 1980s [3]. In fact, a recent small US study using real-world evidence from 308 women found only two trajectories of asthma control, 60% remained the same and 40% had worse control [4]. A much larger Canadian study including exacerbation data between 2005 and 2015 found an increase in hospital admissions and emergency department visits during pregnancy, but the authors were not able to compare the changes across the trimesters, assess the use of oral corticosteroids or investigate the effect of asthma severity and phenotypes [5]. Therefore, it still remains unclear how pregnancy affects a woman's risk of exacerbation.

Asthma exacerbations during pregnancy not only increase the risk of pregnancy complications (*e.g.* pre-eclampsia, gestational hypertension), but are also associated with adverse perinatal and neonatal outcomes (*e.g.* low birthweight, preterm birth, congenital malformations) and early childhood respiratory disorders (*e.g.* asthma, pneumonia) [6–8]. The identification of modifiable risk factors is thus essential to improve care during this critical period. Poor adherence to asthma medication is a key modifiable risk factor, but it is unknown how common it is and to what extent it is associated with pregnancy-related exacerbations. Despite the recognised importance of taking inhaled corticosteroids (ICS) during pregnancy, concerns that asthma medication is unsafe for the fetus, lack of education provision and difficulty in asthma symptom perception during pregnancy may lead to discontinuation of ICS [9–11].

A recent systematic review and meta-analysis of 35 studies investigated risk factors associated with pregnancy-related exacerbations [12]. However, the review found high heterogeneity between studies, with most suffering from small sample size. The findings were also predominantly obtained from prospective pregnancy cohorts, and such studies are at risk of recall and selection bias. There is therefore distinct value in comparing findings from prospective cohorts to those from routinely collected data that represent real-life clinical practice. In addition, there is a lack of understanding of how asthma phenotypes affect a woman's risk during pregnancy.

In this study, we analysed real-world data from the UK to achieve three objectives. First, to understand how pregnancy influences asthma exacerbation patterns. Second, to determine modifiable pregnancy-related exacerbation risk factors. Third, to explore factors associated with reduced ICS use during pregnancy.

## Methods

### Data source

We used the Clinical Practice Research Datalink (CPRD), a nationally representative database of UK primary care electronic healthcare records covering approximately 20% of the population and well-validated for epidemiological research [13, 14]. The CPRD Pregnancy Register contains 16.8 million pregnancy episodes, and each pregnancy record includes details of the start and end of the pregnancy, trimester dates and the outcome of the pregnancy [15, 16]. Individual patient records are linked to the CPRD Pregnancy Register, Hospital Episode Statistics (English hospital admission data) and Office for National Statistics mortality data. All code lists are available on GitHub (https://github.com/BoheeLEE/Pregnancy).

### Study design and population

This longitudinal cohort study included women aged >17 years with asthma, as indicated by at least two asthma codes plus at least one asthma inhaler prescription in the year before pregnancy. Only pregnancies resulting in a live birth between 2004 and 2020 were included; if women had more than one pregnancy, only the latest pregnancy was included (to prevent within-person correlation across multiple pregnancies and to reflect the most recent clinical practices and allow assessment of gravida). Data were obtained from 12 months before until 12 months after pregnancy (supplementary figure E1).

### Outcomes

The primary outcome was asthma exacerbations, defined as a short course (5–7 days) of oral corticosteroids or an unplanned hospital visit for asthma (emergency department, accident and emergency (A&E) or admission: International Classification of Diseases, Tenth Revision, codes J45 and J46).

The secondary outcome was change in frequency of ICS prescriptions during 9 months of pregnancy as compared to the 12 months before pregnancy (“change in ICS”). This was categorised as an increase (a rise of an average rate of ≥0.5 ICS canisters prescribed per year), a decrease (a reduction of an average rate of ≥0.5 ICS canisters prescribed per year) or no change (a change of less than an average rate of ±0.5 ICS canisters prescribed per year). Because an exacerbation may alter the ICS prescription rate, we censored data at the first exacerbation during pregnancy.

### Risk factors

Potential risk factors for pregnancy-related exacerbations were identified through literature review and discussion with clinicians [12, 17]. The following were considered: age, ethnicity, socioeconomic deprivation (measured by quintiles of Index of Multiple Deprivation), general practitioner (GP) practice, body mass index (BMI) (categorised as underweight: ≤18.5 kg·m^−2^; normal: 18.5–24.9 kg·m^−2^; overweight: 25–29.9 kg·m^−2^; obese: ≥30 kg·m^−2^), anxiety/depression, atopy, blood eosinophil count (highest recorded value within 3  years before pregnancy, categorised as <0.3×10^9^ cells·L^−1^ or ≥0.3×10^9^ cells·L^−1^), gravida, smoking habits during pregnancy (never, ex-smoker or current smoker), annual asthma review in the year before pregnancy (review of asthma control and inhaler technique check) and asthma severity/control. Two markers of asthma severity/control were included: medication prescriptions and exacerbations in the year before pregnancy. Medication was categorised into short-acting β-agonists (SABA) only, ICS only or ICS with add-on preventer of long-acting bronchodilator or leukotriene receptor antagonist. ICS prescriptions were further categorised by rate into <4 ICS prescriptions or ≥4 ICS prescriptions per year.

### Statistical analysis

Descriptive statistics were used to present the distribution of asthma exacerbation patterns. In the UK, exacerbations are managed either within primary care (GP practice), or within hospitals through the A&E or by admission to the hospital wards. We calculated the proportion having at least one exacerbation in each 3-month period. Change in inhaler prescriptions, between the different inhaler categories, throughout the follow-up period was illustrated using a Sankey plot.

The association between the potential risk factors and exacerbations during pregnancy was investigated using multivariable logistic regression analysis. In addition, we conducted an analysis for a subgroup including only women at high risk, *i.e.* those who had experienced an exacerbation in the year pre-pregnancy. To examine the association by different management settings (GP-managed or hospital-managed), we performed multinomial logistic regression analysis.

To measure the association between individual characteristics and decrease in ICS during pregnancy, or increase in ICS, we conducted two multivariable logistic regression models.

We modelled missing values as an “unknown” category if they were assumed not to be missing at random, including for BMI, blood eosinophils, smoking and ethnicity. As a sensitivity analysis, for the smoking variable, we imputed missing values using multiple imputation by chained equations; imputation models included all covariates and the outcome, with the results of 10 imputed datasets pooled using Rubin's rules [18].

## Results

The cohort consisted of 40 196 pregnant women with asthma (supplementary figure E2). A total of 4203 women (10.5%) had an exacerbation during pregnancy. A higher proportion of women with a pregnancy-related exacerbation resided in areas of highest deprivation (27.5% *versus* 22.8%), were obese (32.9% *versus* 25.7%), had depression/anxiety (41.9% *versus* 33.2%), had blood eosinophilia (53.4% *versus* 41.0%), were prescribed ICS and add-on therapy (53.9% *versus* 31.5%), had an asthma exacerbation before pregnancy (47.7% *versus* 14.7%) and smoked during pregnancy (24.5% *versus* 16.4%) compared with women with no pregnancy-related exacerbation (table 1).

**TABLE 1:** Cohort characteristics described by asthma exacerbations during pregnancy

Characteristics	All women	No exacerbation during pregnancy	Exacerbation during pregnancy
**Total**	40 196 (100.05)	35 993 (89.5)	4203 (10.5)
**Age group (years)** ^#^			
18–24	14 534 (36.2)	12 929 (35.9)	1605 (38.2)
25–29	11 500 (28.6)	10 297 (28.6)	1203 (28.6)
30–34	9844 (24.5)	8893 (24.7)	951 (22.6)
35–39	3709 (9.2)	3335 (9.3)	374 (8.9)
≥40	609 (1.5)	539 (1.5)	70 (1.7)
**Age (years)**	31.7±5.63	31.6±5.61	31.7±5.79
**Ethnicity**			
White	28 653 (71.3)	25 673 (71.3)	2980 (70.9)
Asian	2498 (6.2)	2182 (6.1)	316 (7.5)
Black	1210 (3.0)	1091 (3.0)	119 (2.8)
Mixed	507 (1.3)	451 (1.3)	56 (1.3)
Other	236 (0.6)	210 (0.6)	26 (0.6)
Unknown	7092 (17.6)	6386 (17.7)	706 (16.8)
**Socioeconomic status (IMD)**			
1 (least deprived)	7603 (18.9)	6927 (19.2)	676 (16.1)
2	7778 (19.4)	7042 (19.6)	736 (17.5)
3	7257 (18.1)	6531 (18.1)	726 (17.3)
4	8210 (20.4)	7302 (20.3)	908 (21.6)
5 (most deprived)	9348 (23.3)	8191 (22.8)	1157 (27.5)
**Body mass index (kg·m^−2^)**			
Underweight (<18.5)	713 (1.8)	641 (1.8)	72 (1.7)
Normal (18.5–24.9)	17 098 (42.5)	15 560 (43.2)	1538 (36.6)
Overweight (24.9–29.9)	11 170 (27.8)	10 005 (27.8)	1165 (27.7)
Obesity (≥30.0)	10 645 (26.5)	9264 (25.7)	1381 (32.9)
Missing	570 (1.4)	523 (1.5)	47 (1.1)
**Smoking**			
Never	15 638 (38.9)	14 141 (39.3)	1497 (35.6)
Ex-smoker	11 467 (28.5)	10 315 (28.7)	1152 (27.4)
Current	11 462 (28.5)	10 043 (27.9)	1419 (33.8)
Missing	1629 (4.1)	1494 (4.2)	135 (3.2)
**Comorbidities**			
Atopy	24 361 (60.6)	21 721 (60.3)	2640 (62.8)
Type 2 diabetes	1470 (3.7)	1259 (3.5)	211 (5.0)
Ischaemic heart disease	241 (0.6)	216 (0.6)	25 (0.6)
Hypertension	788 (2.0)	690 (1.9)	98 (2.3)
GORD	3255 (8.1)	2789 (7.7)	466 (11.1)
PCOS	866 (2.2)	766 (2.1)	100 (2.4)
Depression/anxiety	13 724 (34.1)	11 963 (33.2)	1761 (41.9)
**Blood eosinophil count (×10^9^·L^−1^)**			
Median (IQR)	0.30 (0.20–0.47)	0.30 (0.20–0.45)	0.32 (0.20–0.52)
<0.3	14 643 (36.4)	13 363 (37.1)	1280 (30.5)
≥0.3	17 010 (42.3)	14 765 (41.0)	2245 (53.4)
Missing	8543 (21.3)	7865 (21.9)	678 (16.1)
**Asthma medication (year before pregnancy)** ^¶^			
SABA only	9534 (23.7)	8974 (24.9)	560 (13.3)
ICS <4 prescriptions per year	13 899 (34.6)	12 825 (35.6)	1074 (25.6)
ICS ≥4 prescriptions per year	3020 (7.5)	2738 (7.6)	282 (6.7)
ICS+add-on <4 prescriptions per year	6609 (16.4)	5758 (16.0)	851 (20.2)
ICS+add-on ≥ 4 prescriptions per year	7013 (17.4)	5596 (15.5)	1417 (33.7)
**Asthma exacerbations (year before pregnancy)**			
None	32 910 (81.9)	30 712 (85.3)	2198 (52.3)
1	5104 (12.7)	4098 (11.4)	1006 (23.9)
≥2	2182 (5.4)	1183 (3.3)	999 (23.8)
**Asthma annual review at GP practice**			
Year before pregnancy	11 697 (29.1)	10 430 (29.0)	1267 (30.2)
During pregnancy	12 849 (32.0)	11 311 (31.4)	1538 (36.6)
Year after pregnancy	15 761 (39.2)	13 925 (38.7)	1836 (43.7)
**Pregnancy-related variables**			
Pregnancy length (days)	277.66±10.16	277.69±10.22	277.45±9.59
Multigravida	8893 (22.1)	7809 (21.7)	1084 (25.8)
Smoking during pregnancy			
Yes	6923 (17.2)	5892 (16.4)	1031 (24.5)
No	17 926 (44.6)	16 045 (44.5)	1881 (44.8)
Missing	15 347 (38.2)	14 056 (39.1)	1291 (30.7)

Data are presented as mean±sd or n (%), unless otherwise stated. GORD: gastro-oesophageal reflux disease; GP: general practitioner; ICS: inhaled corticosteroids; IMD: Index of Multiple Deprivation; PCOS: polycystic ovary syndrome; SABA: short-acting β-agonist. ^#^: at the time of pregnancy; ^¶^: “add-on” refers to additional maintenance medication of long-acting β-agonist or leukotriene receptor antagonists.

### Exacerbation patterns

**FIGURE 1:**
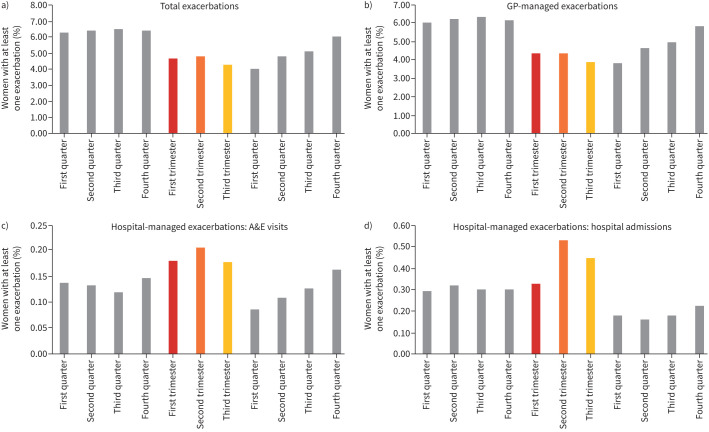
Proportion of women with at least one exacerbation during each 3-month time period for a) total exacerbations, b) general practitioner (GP)-managed exacerbations and c, d) hospital-managed exacerbations *via* accident and emergency department (A&E) visits (c) and hospital admissions (d).

Exacerbations decreased during pregnancy and gradually increased back to pre-pregnancy levels within 9–12 months post-pregnancy (figure 1). Exacerbations decreased by 28% in the first trimester, 28% in the second trimester and 36% in the third trimester. When stratified by management setting, this pattern was driven predominantly by GP-managed exacerbations, which accounted for around 95% of all cases, whereas A&E visits and hospital admissions both increased during pregnancy. A&E visits increased in all three trimesters, by approximately 40%, but fell to low levels postpartum (first quarter post-pregnancy). Hospital admissions increased only during the second and third trimesters, by approximately 43%, and fell considerably postpartum by around 57%, remaining lower throughout the post-pregnancy year.

**FIGURE 2:**
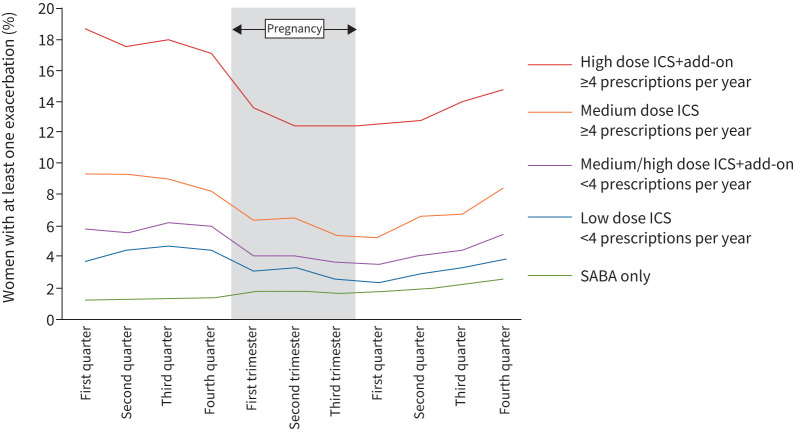
Proportion of women with exacerbations, by their baseline asthma severity. ICS: inhaled corticosteroid; SABA: short-acting β-agonist.

When stratified by asthma severity, the most dramatic fall in pregnancy-related exacerbations was observed in women with the most severe asthma (figure 2).

### Inhaler prescriptions during pregnancy

**FIGURE 3:**
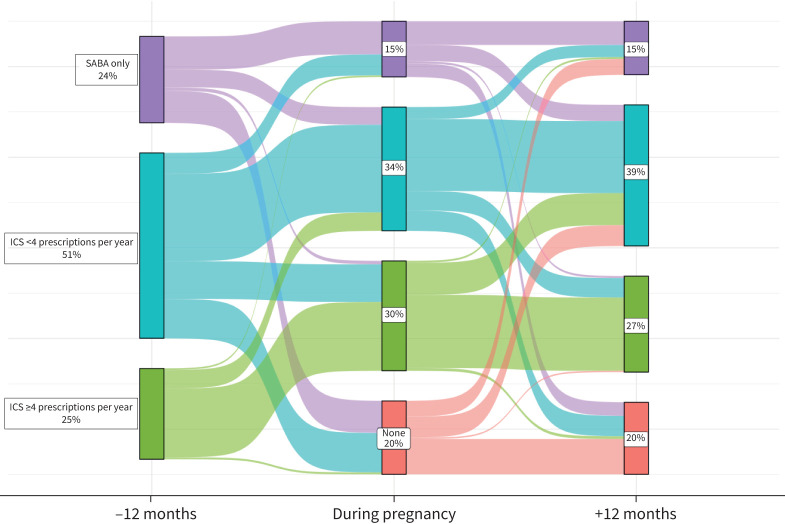
Change in asthma inhaler use during and after pregnancy (n=39 985). ICS: inhaled corticosteroid; SABA: short-acting β-agonist.

Although many women did not change prescription frequency during pregnancy, 31% (12 556) reduced prescriptions (figure 3). One in five women had no inhalers prescribed during pregnancy, of whom nearly half (48%, 3901), remained off inhalers postpartum. Encouragingly, few of the women on ≥4 ICS prescriptions per year pre-pregnancy completely stopped ICS prescriptions during pregnancy. Around one in 10 (13%, 5096) increased medication during pregnancy, increasing ICS frequency or changing from SABA only to ICS prescriptions.

### Association between maternal characteristics and exacerbations during pregnancy

**FIGURE 4:**
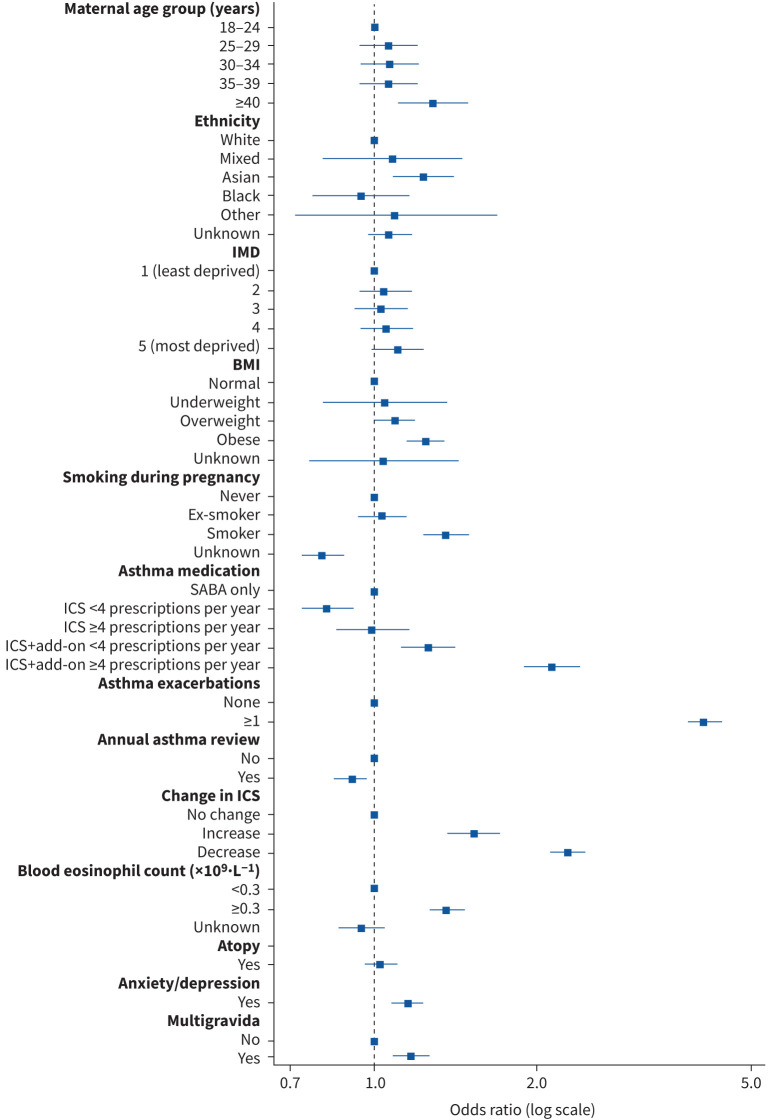
Forest plot for the association between maternal characteristics and asthma exacerbations during pregnancy. BMI: body mass index; ICS: inhaled corticosteroid; IMD: Index of Multiple Deprivation; SABA: short-acting β-agonist.

The three risk factors associated with the highest odds of an exacerbation during pregnancy were decreased use of ICS during pregnancy (adjusted odds ratio (aOR) 2.29, 95% CI 2.12–2.47), exacerbation in the year before pregnancy (aOR 4.09, 95% CI 3.81–4.39) and the highest level of asthma medication in the year before pregnancy (ICS+add-on ≥4 prescriptions per year) (aOR 2.13, 95% CI 1.89–2.40) (supplementary table E1 and figure 4). Other significantly associated factors included older maternal age (≥40 years) (aOR 1.29, 95% CI 1.10–1.50), Asian ethnicity (aOR 1.24, 95% CI 1.08–1.42), overweight (aOR 1.09, 95% CI 1.00–1.19), obese (aOR 1.24, 95% CI 1.14–1.35), current smoker (aOR 1.36, 95% CI 1.23–1.51), ICS+add-on <4 prescriptions per year (aOR 1.26, 95% CI 1.11–1.43), blood eosinophilia (aOR 1.36, 95% CI 1.26–1.47) and comorbid anxiety/depression (aOR 1.15, 95% CI 1.07–1.24).

An annual asthma review in the year before pregnancy was associated with a small but significant decrease in odds of having an exacerbation (aOR 0.91, 95% CI 0.84–0.98).

In a sensitivity analysis using multiple imputation for smoking during pregnancy, the results remained consistent (supplementary table E2).

In the stratified analysis only including women with a history of exacerbations before pregnancy, the biggest risk factor for exacerbations was reducing ICS prescriptions (aOR 2.48, 95% CI 2.30–2.68) (supplementary figure E3).

### Association between maternal characteristics and exacerbations, according to where the exacerbation was managed

When considering where the exacerbation was managed, most factors remained similar in direction and strength as in the main analysis (table 2). Decreasing ICS use during pregnancy was more strongly associated with hospital-managed exacerbations (aOR 2.60, 95% CI 2.13–3.16) than GP-managed exacerbations (aOR 1.92, 95% CI 1.74–2.13). Two factors were only associated with GP-managed exacerbations: maternal age ≥40 years (aOR 1.38, 95% CI 1.13–1.68) and multigravida (aOR 1.17, 95% CI 1.06–1.30). Three factors were only associated with hospital-managed exacerbations: Black ethnicity (aOR 2.00, 95% CI 1.33–3.02), the highest social deprivation (aOR 1.39, 95% CI 1.04–1.87) and a history of previous smoking (aOR 1.55, 95% CI 1.21–1.98).

**TABLE 2:** Multinomial logistic regression measuring the association between maternal characteristics and asthma exacerbations during pregnancy by where the exacerbation was managed (GP *versus* hospital)

	None	≥1 OCS by GP		≥1 hospital visit^#^	
		Adjusted OR (95% CI)	p-value	Adjusted OR (95% CI)	p-value
**Age group (years)**					
18–24	-	Ref		Ref	
25–29	-	1.11 (0.95–1.31)	0.189	1.11 (0.84–1.46)	0.469
30–34	-	1.15 (0.99–1.35)	0.072	0.81 (0.61–1.07)	0.138
35–39	-	1.15 (0.98–1.36)	0.086	0.80 (0.60–1.08)	0.149
≥40	-	1.38 (1.13–1.68)	0.001	0.70 (0.47–1.05)	0.088
**Ethnicity**					
White	-	Ref		Ref	
Mixed	-	0.87 (0.58–1.32)	0.510	1.49 (0.76–2.92)	0.241
Asian	-	1.24 (1.05–1.47)	0.013	1.56 (1.13–2.16)	0.008
Black	-	0.80 (0.60–1.07)	0.137	2.00 (1.33–3.02)	0.001
Others	-	1.17 (0.70–1.96)	0.541	NA (not converged)	NA
Unknown	-	1.01 (0.90–1.14)	0.867	1.13 (0.9–1.41)	0.312
**Socioeconomic status (IMD)**					
1 (least deprived)	-	Ref		Ref	
2	-	0.94 (0.81–1.09)	0.398	1.33 (0.98–1.82)	0.069
3	-	0.97 (0.84–1.12)	0.714	1.24 (0.91–1.70)	0.170
4	-	1.02 (0.89–1.17)	0.792	1.24 (0.91–1.68)	0.168
5 (most deprived)	-	1.02 (0.89–1.17)	0.802	1.39 (1.04–1.87)	0.028
**Body mass index (kg·m^−2^)**					
Normal	-	Ref		Ref	
Underweight		0.92 (0.64–1.32)	0.646	0.97 (0.49–1.90)	0.927
Overweight	-	1.10 (0.99–1.23)	0.071	1.03 (0.83–1.29)	0.757
Obese	-	1.30 (1.17–1.45)	<0.001	1.29 (1.05–1.59)	0.014
Unknown	-	1.13 (0.75–1.69)	0.560	1.14 (0.52–2.51)	0.739
**Smoking during pregnancy**					
Never	-	Ref		Ref	
Ex-smoker	-	1.03 (0.90–1.17)	0.682	1.55 (1.21–1.98)	0.001
Current smoker	-	1.35 (1.18–1.53)	<0.001	1.36 (1.05–1.75)	0.019
Unknown	-	0.81 (0.73–0.91)	<0.001	0.79 (0.62–1.00)	0.050
**Asthma medications (year before pregnancy)**					
SABA only	-	Ref		Ref	
ICS <4 prescriptions per year	-	0.99 (0.86–1.14)	0.926	0.67 (0.49–0.92)	0.014
ICS ≥4 prescriptions per year	-	1.13 (0.92–1.37)	0.242	0.82 (0.54–1.26)	0.366
ICS+add-on <4 prescriptions per year	-	1.41 (1.21–1.64)	<0.001	1.39 (1.01–1.92)	0.041
ICS+add-on ≥ 4 prescriptions per year	-	2.25 (1.94–2.61)	<0.001	2.16 (1.59–2.94)	<0.001
**Asthma exacerbations (year before pregnancy)**					
None		Ref		Ref	
≥1		6.73 (5.99–7.57)	<0.001	16.55 (13.78–19.87)	<0.001
**Asthma annual review (year before pregnancy)**					
None					
Yes		0.95 (0.87–1.05)	0.343	0.87 (0.72–1.05)	0.156
**Change in ICS during pregnancy**					
No change	-	Ref		Ref	
Increase ICS	-	1.68 (1.46–1.93)	<0.001	1.33 (0.97–1.83)	0.081
Decrease ICS	-	1.92 (1.74–2.13)	<0.001	2.60 (2.13–3.16)	<0.001
**Blood eosinophil count before pregnancy (×10^9^·L^−1^)**					
<0.3	-	Ref		Ref	
≥0.3	-	1.29 (1.17–1.42)	<0.001	1.43 (1.17–1.76)	<0.001
Unknown	-	0.82 (0.72–0.94)	0.004	1.2 (0.93–1.55)	0.156
**Atopy**					
No		Ref		Ref	
Yes	-	1.04 (0.95–1.13)	0.433	1.02 (0.85–1.22)	0.813
**Anxiety/depression**					
No	-	Ref		Ref	
Yes	-	1.19 (1.08–1.30)	<0.001	1.27 (1.06–1.51)	0.009
**Multigravida**					
No	-	Ref		Ref	
Yes	-	1.17 (1.06–1.30)	0.002	1.06 (0.86–1.3)	0.594

GP: general practitioner; ICS: inhaled corticosteroids; IMD: Index of Multiple Deprivation; NA: not available; OCS: oral corticosteroids; SABA: short-acting β-agonists.

^#^: hospital visits include accident and emergency department visits and hospitalisation.

### Associations between maternal characteristics and pregnancy-related change in ICS prescriptions

**FIGURE 5:**
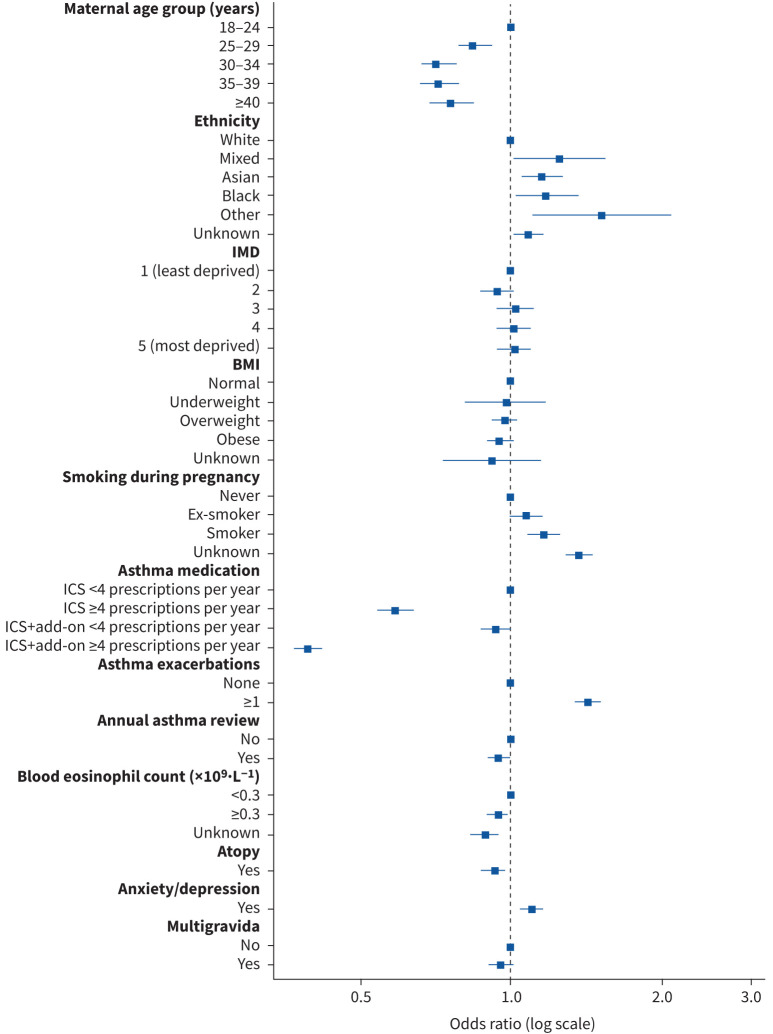
Forest plot for the association between maternal characteristics and decreased inhaled corticosteroid (ICS) use during pregnancy. BMI: body mass index; IMD: Index of Multiple Deprivation.

Several factors were significantly associated with decreased ICS prescriptions during pregnancy. These included non-White ethnicity, history of smoking, smoking during pregnancy and anxiety/depression (supplementary table E3 and figure 5). Factors significantly associated with not decreasing ICS were age >25 years, ICS ≥4 prescriptions per year and atopy.

Factors significantly associated with increased ICS prescriptions included age >35 years, obesity and blood eosinophilia (supplementary table E3).

## Discussion

In this nationwide cohort, we observed that around 10% of women experienced asthma exacerbations during pregnancy, a similar proportion to that found in Australian asthma pregnancy cohorts, but almost double that found in a Canadian population-based cohort [6, 17]. Total exacerbations decreased during pregnancy, driven by an approximate 30% reduction in exacerbations managed in primary care. In contrast, exacerbations requiring acute hospital management increased, by up to 40%, particularly in the second and third trimesters. This pattern broadly correlates with the only other large real-world study, from Canada [5]. However, the Canadian study was unable to address factors influencing exacerbations owing to a lack of access to information on asthma severity, medication, smoking, ethnicity and eosinophil counts.

The change in exacerbations during pregnancy is likely multifactorial. Hormonal changes may contribute; however, that would not explain the discordant trends in exacerbations managed across different healthcare settings. Furthermore, prior studies suggest that the impact of hormonal fluctuations on exacerbation risk is marginal [19, 20]. Healthcare behaviour could be a critical factor. Some women may improve adherence to ICS and adopt trigger-avoidance strategies during pregnancy, reducing their exacerbation risk. Conversely, others may discontinue medication due to concerns about fetal safety [21]. Additionally, reluctance to initiate oral corticosteroids during pregnancy may contribute to suboptimal management of worsening symptoms. The observed increase in hospital visits could reflect more severe asthma symptoms coinciding with pregnancy-related dyspnoea or heightened maternal and physician concern for fetal wellbeing and a more risk-averse management approach, leading to a preference for hospital-based care. Some women may also seek hospital care in anticipation of additional pregnancy-related assessments. Alternatively, altered antiviral immunity during pregnancy could increase susceptibility to respiratory infections, leading to more severe exacerbations requiring hospitalisation. Postpartum, the marked and persistent reduction in hospital visits could be attributed to changes in lung volumes, hormonal shifts or practical constraints, such as reduced capacity for a new mother to seek hospital care.

The strongest predictors of pregnancy-related exacerbations were poor asthma control pre-pregnancy (pre-pregnancy exacerbations quadrupled the odds), reduced ICS during pregnancy (more than doubling the odds) and greater asthma severity (the highest level of pre-pregnancy asthma medication doubled the odds). Our pre-pregnancy exacerbation findings are consistent with two previous, smaller studies [12]. However, our finding regarding reduced ICS contrasts with a Canadian medication trajectory study, which found increased ICS during pregnancy to be the greatest risk factor [22]. But, as the authors acknowledged, their findings were likely influenced by including prescriptions after exacerbations. To avoid reverse causation, we only included prescriptions prior to exacerbations (censoring on exacerbations). Additionally, among the highest-risk women (those with a pre-pregnancy exacerbation), the single biggest predictor of a pregnancy-related exacerbation was reducing ICS.

In our cohort, approximately one in three women reduced their inhaler use. This shift may be driven by limited awareness of the benefits of asthma medication during pregnancy [11], concerns about potential risks to the fetus [21] or suboptimal self-management skills [9, 23]. This issue may also be attributable to clinician practices. An Australian survey found that a quarter of GPs would stop or reduce a woman's ICS during pregnancy [24], underscoring the need for improved education for patients and healthcare providers.

Some women discontinued inhaler prescriptions entirely and remained off them in the year postpartum. This suggests either that there was spontaneous remission of previously active asthma or a misdiagnosis of asthma, supporting studies that indicate a high prevalence of overtreatment and misdiagnosis in the community [25, 26].

By contrast, one in ten women increased their ICS prescriptions, nearly half of whom then reduced ICS use postpartum. This pregnancy-related increase may have been due to worsening asthma, pregnancy-induced dyspnoea, which can be difficult to distinguish from asthma symptoms, or heightened vigilance in managing asthma to optimise maternal and neonatal health.

Other risk factors for exacerbations during pregnancy included older maternal age, Asian ethnicity, elevated BMI, smoking during pregnancy, anxiety or depression, and multigravida. These findings align with those from a meta-analysis involving over 400 000 pregnant women [12]. However, the meta-analysis included only two studies with ethnicity data, both conducted in the US [27, 28]. The US studies reported a 62% increased risk of exacerbations among Black pregnant women. By contrast, our study found 24% increased odds among women of Asian ethnicity, but no significant association with Black ethnicity. This discrepancy is likely explained by differences in healthcare utilisation, because pregnant women of Black ethnicity in our study had 20% reduced odds of a GP-managed exacerbation but double the odds of a hospital-managed exacerbation. Variations in healthcare systems, as well as racial and ethnic disparities in access to care, asthma management and medication perceptions during pregnancy, may contribute to these differences [29].

The association between elevated BMI and pregnancy-related exacerbations suggests that weight loss interventions should be considered in preconception care [30, 31]. Incorporating weight management strategies, such as tailored dietary advice, physical activity programmes and behavioural support, may offer dual benefits of improving asthma outcomes and supporting a healthy pregnancy [32]. Furthermore, the growing evidence for obesity medication, including glucagon-like peptide-1 receptor agonists, reducing exacerbations warrants carefully designed trials in women with asthma who are planning pregnancy [33, 34].

Few studies have examined the role of pre-pregnancy blood eosinophil count as a biomarker for pregnancy-related exacerbations. We found that elevated eosinophil counts (using a modest threshold of 0.3×10^9^ cells·L^−1^) were associated with 36% increased odds, a risk comparable to smoking during pregnancy. However, the role of biomarkers in pregnancy remains uncertain. A randomised controlled trial in pregnancy using exhaled nitric oxide fraction-guided therapy reduced exacerbations compared to a symptom-based approach [35]; however, *post hoc* analysis showed no reduction in eosinophilic exacerbations [36]. This highlights the need for further validation of biomarker-directed strategies in pregnancy.

We found dedicated primary care asthma reviews were associated with a modest but significant reduction in pregnancy exacerbations, particularly hospital-managed exacerbations. In the UK, an asthma review should include checking inhaler technique, asthma control, exacerbations and provision of a self-management plan. Our results are encouraging and reinforce the importance of focusing on core asthma care when planning for pregnancy. Previous studies have highlighted a lack of asthma self-management skills in pregnant women, implying potential for improving outcomes [9].

Because reducing ICS was a major modifiable exacerbation risk factor, we investigated factors associated with this pattern. Several demographic and clinical characteristics demonstrated modest associations, including younger maternal age (<25 years), non-White ethnicity, a history of current or past smoking, and anxiety or depression. However, the strongest associations were observed in relation to markers of suboptimal asthma control pre-pregnancy (pre-pregnancy exacerbations and pre-pregnancy low use of preventer medication). This further highlights the importance of optimising asthma management pre-pregnancy.

Our study has several strengths. We used a large, nationally representative, longitudinal dataset, which represents real-world clinical practice and included details on prescriptions and blood eosinophil count. The use of routinely collected data reduces the risk of recall and selection bias and increases the generalisability of our results. There are several limitations. We do not know if the medication was dispensed or adhered to. We do not know why prescription frequency changed during pregnancy; therefore, we assessed the factors that were associated with changes in prescription frequency. Some variables, such as asthma control questionnaire scores and spirometry values, were not available, and due to the lack of objective test results, asthma may have been misdiagnosed in some women. We only included pregnancies resulting in a live birth and that had at least 1 year of data pre-pregnancy and postpartum.

### Conclusions

In this population-based study of pregnant women with asthma we found that, despite a decrease in exacerbations managed in primary care, emergency hospital visits for asthma exacerbations increased during pregnancy. This study reaffirms the importance of addressing modifiable pregnancy risk factors, such as smoking and elevated BMI, optimising pre-pregnancy asthma control, providing education, and supporting pregnant women in adhering to ICS. Clinicians should be particularly vigilant in identifying high-risk phenotypes: women with suboptimally controlled asthma, type 2 high asthma and those with poor ICS adherence during pregnancy.

## Shareable PDF

10.1183/13993003.01327-2025.Shareable1 This PDF extract can be shared freely online.
Shareable PDF

ERJ-01327-2025.Shareable

## Data Availability

This study used anonymised electronic health records, CPRD, obtained under license from the UK Medicines and Healthcare Products Regulatory Agency. According to the UK Data Protection Act, electronic health records are regarded as “sensitive data”, which prevents data sharing *via* public deposition. To access CPRD data and linked data such as Hospital Episodes Statistics, data from the Office for National Statistics and Index of Multiple Deprivation data requires approval *via* CPRD's Research Data Governance process (https://cprd.com/data-access). Data management was provided by the Big Data and Analytical Unit at the Institute of Global Health Innovation.

## Supplementary material

**Please note:** supplementary material is not edited by the Editorial Office, and is uploaded as it has been supplied by the author. Supplementary material ERJ-01327-2025.Supplement
