# Supplementary material for: Pregnancy, asthma and exacerbations: a population-based cohort
Source: Eur Respir J. 2025 Dec 18;66(6):2501327. doi: 10.1183/13993003.01327-2025 (PMC12713386; doi:10.1183/13993003.01327-2025)
Supplement: Supplementary file 1 [file ERJ-01327-2025.Shareable.pdf]

EUROPEAN RESPIRATORY JOURNAL
ORIGINAL RESEARCH ARTICLE
B. LEE ET AL.

SHAREABLE PDF

Pregnancy, asthma and exacerbations: a population-based cohort

Bohee Lee, Ernie Wong, Tricia Tan, Hitasha Rupani and Chloe I. Bloom

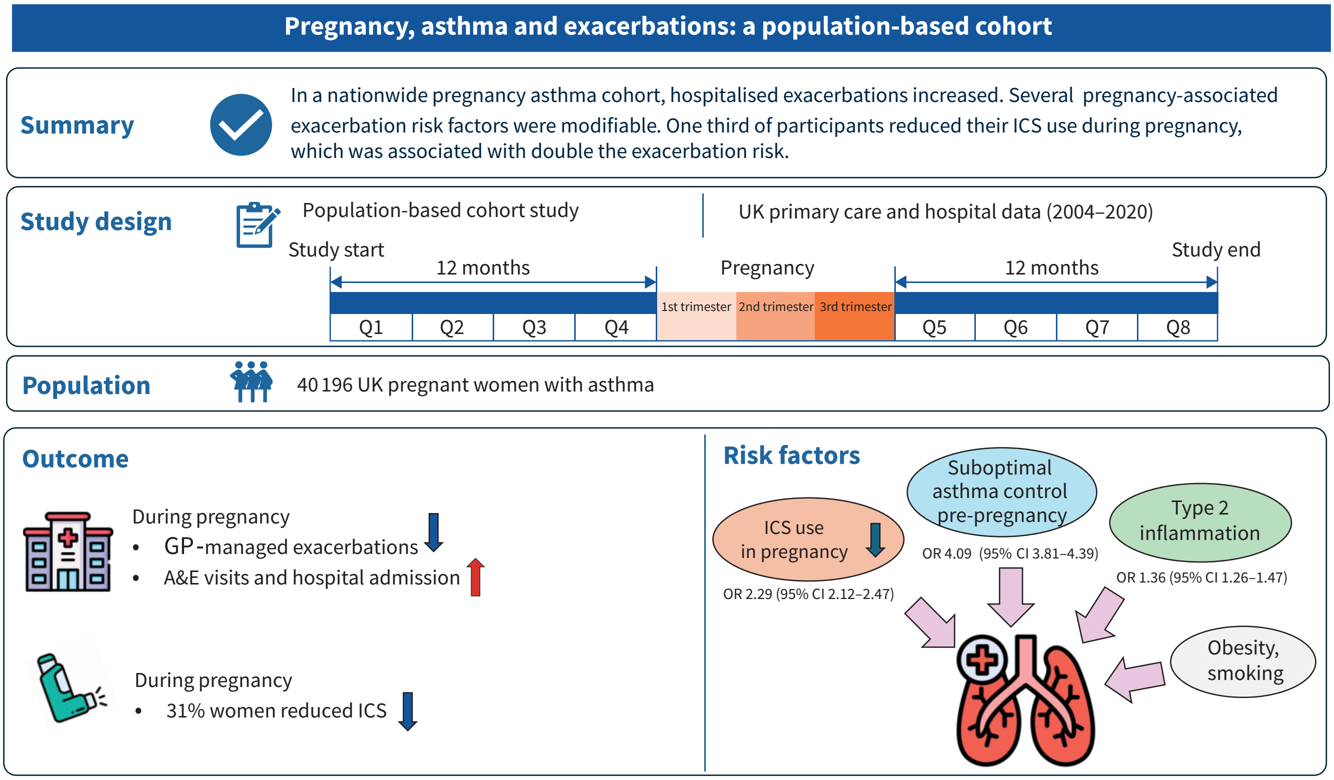

GRAPHICAL ABSTRACT Overview of the study. A&E: accident and emergency department; GP: general practitioner; ICS: inhaled corticosteroids.

EUROPEAN RESPIRATORY JOURNAL
ORIGINAL RESEARCH ARTICLE
B. LEE ET AL.

SHAREABLE PDF

Pregnancy, asthma and exacerbations: a population-based cohort

Bohee Lee [1], Ernie Wong[1], Tricia Tan[2], Hitasha Rupani [3] and Chloe I. Bloom [1]

[1]National Heart and Lung Institute, Imperial College London, London, UK. [2]Department of Metabolism, Digestion and Reproduction, Imperial College London, London, UK. [3]Department of Respiratory Medicine, University Hospitals Southampton NHS Foundation Trust, Southampton, UK.

Corresponding author: Chloe I. Bloom (chloe.bloom06@imperial.ac.uk)

Check for updates

Shareable abstract (@ERSpublications)
In a large pregnancy asthma cohort, hospitalised exacerbations increased. Many risk factors were modifiable. A third of women reduced their inhaled steroid prescriptions during pregnancy, doubling exacerbation risk. Other factors included suboptimal control and type 2 inflammation. https://bit.ly/3Jri7OL

Cite this article as: Lee B, Wong E, Tan T, *et al*. Pregnancy, asthma and exacerbations: a population-based cohort. *Eur Respir J* 2025; 66: 2501327 [DOI: 10.1183/13993003.01327-2025].

This PDF extract can be shared freely online.

Copyright ©The authors 2025

This version is distributed under the terms of the Creative Commons Attribution Licence 4.0.

This article has an editorial commentary: https://doi.org/10.1183/13993003.01979-2025

Received: 17 April 2025
Accepted: 14 Aug 2025

Abstract

Background Asthma exacerbations during pregnancy are associated with adverse maternal and perinatal outcomes. Identifying modifiable risk factors is essential for improving health outcomes. We aimed to describe exacerbation patterns during pregnancy and identify exacerbation risk factors, particularly modifiable risk factors such as inhaled corticosteroid use.

Methods This was a cohort study using UK primary care and hospital data (2004–2020) to identify pregnant women with asthma. Exacerbations were defined as a short course of oral corticosteroids, emergency department visit or unscheduled hospital admission. Multivariable logistic regression was used to assess associations between maternal characteristics and exacerbations (primary outcome) and inhaled corticosteroid use (secondary outcome).

Results Among 40 196 pregnant women with asthma, total exacerbations declined by ∼30% during pregnancy. However, exacerbations associated with hospital admission increased by 30–45% during the second and third trimesters, declining abruptly after delivery. Inhaled corticosteroid prescriptions were reduced in 31% of women during pregnancy. Decreased inhaled corticosteroid use was associated with suboptimal asthma control pre-pregnancy, age, ethnicity and smoking. The strongest exacerbation risk factors were a history of exacerbations (adjusted OR 4.09, 95% CI 3.81–4.39), reduced inhaled corticosteroid use during pregnancy (adjusted OR 2.29, 95% CI 2.12–2.47) and ⩾4 prescriptions per year for inhaled corticosteroids plus another preventer before pregnancy (adjusted odds ratio 2.11, 95% CI 1.87–2.37). Additional risk factors included blood eosinophilia, smoking and obesity.

Conclusions Despite fewer total exacerbations, exacerbations associated with a hospital admission increased during pregnancy. One third of women reduced inhaled corticosteroid use during pregnancy, yet this was the second largest exacerbation risk factor and is completely modifiable. Other major risk factors were type 2 inflammation and another modifiable risk factor, suboptimal asthma control pre-pregnancy.
