# Supplementary material for: Pregnancy, asthma and exacerbations: a population-based cohort
Source: Eur Respir J. 2025 Dec 18;66(6):2501327. doi: 10.1183/13993003.01327-2025 (PMC12713386; doi:10.1183/13993003.01327-2025)
Supplement: Supplementary file 2 [file ERJ-01327-2025.Supplement.pdf]

Pregnancy, asthma and exacerbations: a population-based cohort

Contents

Figure E 1. Diagram of study design 2

Figure E 2. Flow chart of cohort selection 3

Figure E 3. Forest plot for the associations between maternal characteristics and asthma exacerbation during pregnancy in women having a history of previous asthma exacerbations 4

Table E 1. Multivariable logistic regression measuring the association between maternal characteristics and asthma exacerbations during pregnancy 5

Table E 2. Sensitivity analysis applying multiple imputation for smoking during pregnancy 7

Table E 3. Logistic regression measuring the association between maternal characteristics and change in ICS during pregnancy 9

Figure E 1. Diagram of study design

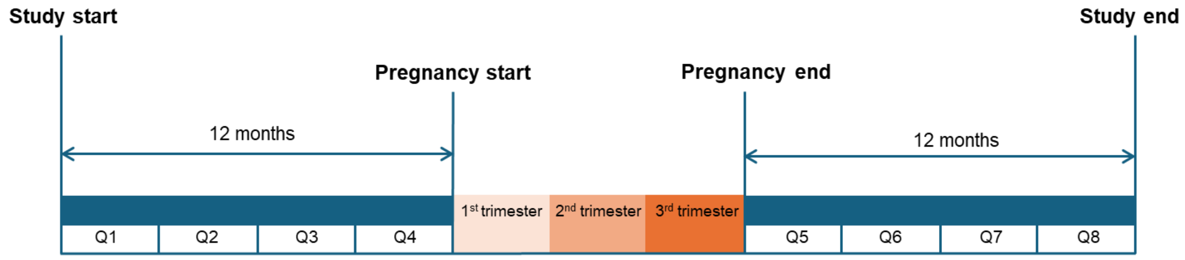

Q1~4: a 3-month period before pregnancy; Q5~8: a 3-month period after pregnancy

Figure E 2. Flow chart of cohort selection

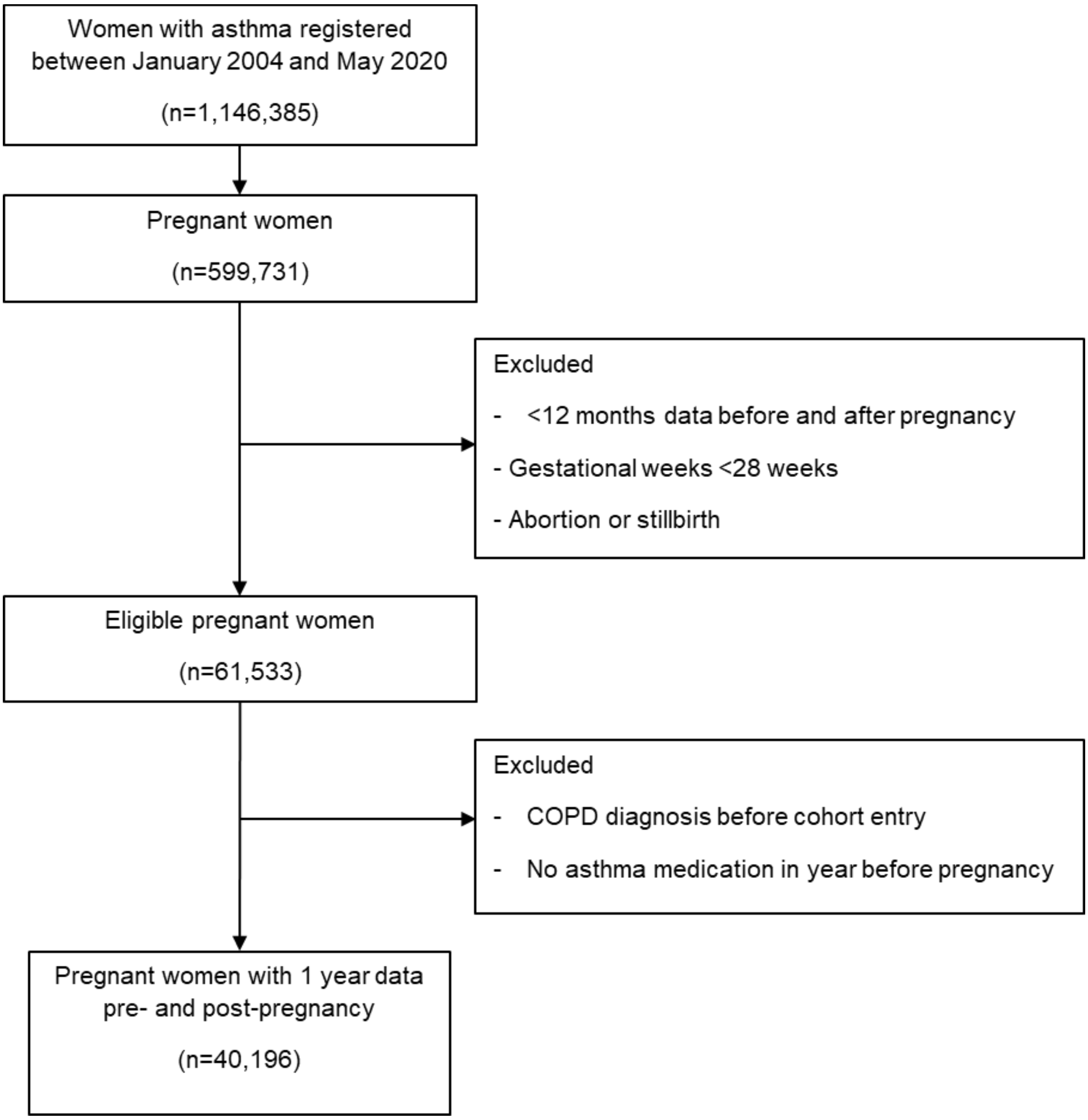

Figure E 3. Forest plot for the associations between maternal characteristics and asthma exacerbation during pregnancy in women having a history of previous asthma exacerbations

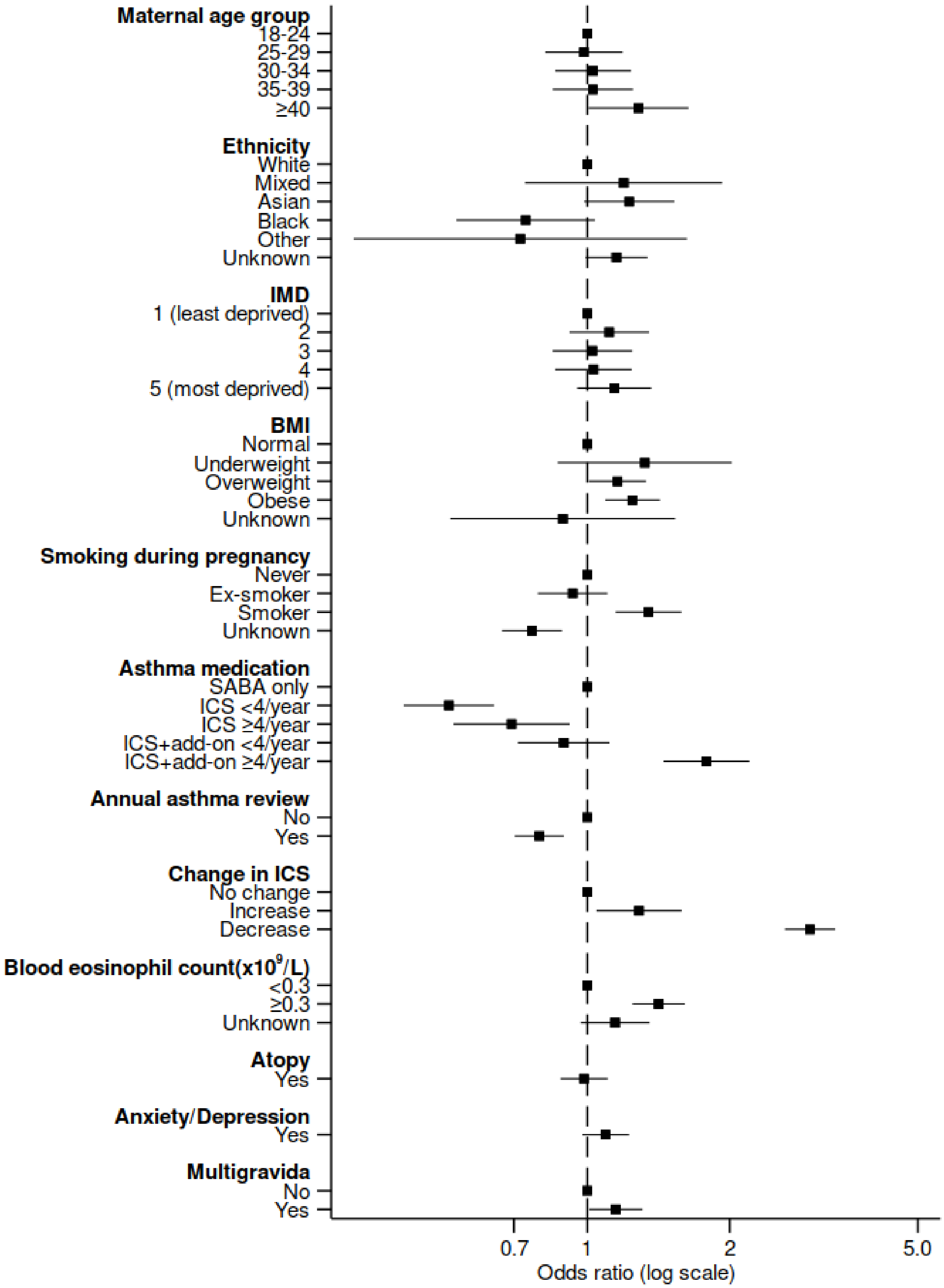

Table E 1. Multivariable logistic regression measuring the association between maternal characteristics and asthma exacerbations during pregnancy

	Adjusted OR (95% CI)	p-value
**Maternal age group**		
18–24 yr	Ref	
25–29 yr	1.06 [0.94-1.20]	0.342
30–34 yr	1.06 [0.95-1.20]	0.305
35–39 yr	1.07 [0.94-1.21]	0.317
≥40y	1.29 [1.10-1.50]	0.001
**Ethnicity**		
White	Ref	
Mixed	1.08 [0.80-1.46]	0.612
Asian	1.24 [1.08-1.42]	0.002
Black	0.94 [0.76-1.16]	0.569
Others	1.09 [0.71-1.70]	0.688
Unknown	1.06 [0.97-1.17]	0.187
**IMD**		
1 (least deprived)	Ref	
2	1.05 [0.93-1.18]	0.443
3	1.03 [0.92-1.16]	0.627
4	1.06 [0.94-1.18]	0.351
5	1.11 [0.99-1.24]	0.078
**BMI** (kg/m^2)		
Normal	Ref	
Underweight	1.05 [0.80-1.37]	0.727
Overweight	1.09 [1.00-1.19]	0.045
Obese	1.24 [1.14-1.35]	<0.001
Unknown	1.04 [0.75-1.43]	0.811
**Smoking during pregnancy**		
Never	Ref	
Ex-smoker	1.03 [0.93-1.14]	0.561
Current smoker	1.36 [1.23-1.51]	<0.001
Unknown	0.80 [0.73-0.88]	<0.001
**Asthma medication (year before pregnancy)**		
SABA only	Ref	
ICS <4/year	0.82 [0.73-0.92]	0.001
ICS ≥4/year	0.99 [0.84-1.16]	0.908
ICS + add-on <4/year	1.26 [1.11-1.43]	<0.001
ICS + add-on ≥4/year	2.13 [1.89-2.40]	<0.001
**Asthma exacerbations (year before pregnancy)**		
None	Ref	
≥1	4.09 [3.81-4.39]	<0.001
**Annual asthma review (year before pregnancy)**		
No	Ref	
Yes	0.91 [0.84-0.98]	0.010
**Change in ICS during pregnancy**		
No change	Ref	
Increase ICS	1.53 [1.36-1.72]	<0.001
Decrease ICS	2.29 [2.12-2.47]	<0.001

	Adjusted OR (95% CI)	p-value
**Blood eosinophil count before pregnancy ($x10^9$/L)**		
<0.3	Ref	
≥0.3	1.36 [1.26-1.47]	<0.001
Unknown	0.95 [0.85-1.05]	0.289
**Atopy**		
No	Ref	
Yes	1.02 [0.95-1.10]	0.534
**Anxiety/Depression**		
No	Ref	
Yes	1.15 [1.07-1.24]	<0.001
**Multigravida**		
No	Ref	
Yes	1.17 [1.08-1.27]	<0.001

Abbreviation: OR=odds ratio, 95%CI= 95% confidence interval, IMD= Index of Multiple Deprivation, BMI=body mass index (kg/m^2), ICS= Inhaled Corticosteroids, SABA= Short-Acting Beta-Agonists

Table E 2. Sensitivity analysis applying multiple imputation for smoking during pregnancy

	Adjusted OR (95% CI)	p-value
**Maternal age group**		
18–24 yr	Ref	
25–29 yr	1.03 [0.92-1.17]	0.595
30–34 yr	1.02 [0.91-1.15]	0.694
35–39 yr	1.02 [0.90-1.16]	0.724
≥40y	1.23 [1.06-1.44]	0.007
**Ethnicity**		
White	Ref	
Mixed	1.06 [0.79-1.43]	0.706
Asian	1.18 [1.03-1.36]	0.015
Black	0.92 [0.75-1.13]	0.439
Others	1.06 [0.69-1.65]	0.778
Unknown	1.05 [0.96-1.15]	0.302
**IMD**		
1 (least deprived)	Ref	
2	1.05 [0.93-1.17]	0.449
3	1.04 [0.92-1.17]	0.528
4	1.07 [0.96-1.20]	0.219
5	1.13 [1.01-1.27]	0.029
**BMI (kg/m^2)**		
Normal	Ref	
Underweight	1.04 [0.80-1.36]	0.749
Overweight	1.09 [1.00-1.19]	0.049
Obese	1.24 [1.14-1.35]	<0.001
Unknown	1.01 [0.74-1.40]	0.935
**Smoking during pregnancy**		
Never	Ref	
Ex-smoker	1.03 [0.93-1.15]	0.524
Current smoker	1.24 [1.13-1.36]	<0.001
**Asthma medication**		
SABA only	Ref	
ICS <4/year	0.83 [0.74-0.93]	0.002
ICS ≥4/year	1.02 [0.87-1.20]	0.775
ICS + add-on <4/year	1.28 [1.13-1.45]	<0.001
ICS + add-on ≥4/year	2.21 [1.96-2.49]	<0.001
**Asthma exacerbations**		
None	Ref	
≥1	4.09 [3.81-4.39]	<0.001
**Annual asthma review before pregnancy**		
No	Ref	
Yes	0.90 [0.83-0.97]	0.006
**Change in ICS during pregnancy**		
No change	Ref	
Increase ICS	1.58 [1.41-1.77]	<0.001
Decrease ICS	2.26 [2.09-2.44]	<0.001
**Blood eosinophil count (x10^9/L)**		
<0.3	Ref	

	Adjusted OR (95% CI)	p-value
≥0.3	1.36 [1.25-1.47]	<0.001
Unknown	0.95 [0.85-1.05]	0.294
**Atopy**		
No	Ref	
Yes	1.02 [0.95-1.10]	0.564
**Anxiety/Depression**		
No	Ref	
Yes	1.17 [1.08-1.25]	<0.001
**Multigravida**		
No	Ref	
Yes	1.16 [1.07-1.26]	<0.001

Abbreviation: OR=odds ratio, 95%CI= 95% confidence interval, IMD= Index of Multiple Deprivation, BMI=body mass index (kg/m^2), ICS= Inhaled Corticosteroids, SABA= Short-Acting Beta-Agonists

Table E 3. Logistic regression measuring the association between maternal characteristics and change in ICS during pregnancy

	Increased ICS		Decreased ICS	
	Adjusted OR (95% CI)	p-value	Adjusted OR (95% CI)	p-value
**Maternal age group**				
18–24 yr	Ref		Ref	
25–29 yr	1.05 [0.94-1.18]	0.366	0.85 [0.77-0.92]	<0.001
30–34 yr	1.07 [0.96-1.19]	0.250	0.72 [0.66-0.78]	<0.001
35–39 yr	1.16 [1.03-1.30]	0.015	0.72 [0.66-0.79]	<0.001
≥40y	1.18 [1.01-1.37]	0.034	0.76 [0.68-0.85]	<0.001
**Ethnicity**				
White	Ref		Ref	
Mixed	0.79 [0.57-1.08]	0.136	1.25 [1.01-1.54]	0.039
Asian	1.05 [0.92-1.21]	0.441	1.15 [1.04-1.27]	0.006
Black	0.93 [0.77-1.12]	0.417	1.18 [1.02-1.36]	0.024
Others	1.67 [1.13-2.47]	0.009	1.52 [1.10-2.10]	0.010
Unknown	0.96 [0.89-1.05]	0.390	1.09 [1.02-1.16]	0.015
**IMD**				
1 (least deprived)	Ref		Ref	
2	0.92 [0.83-1.02]	0.115	0.94 [0.87-1.02]	0.135
3	0.96 [0.87-1.07]	0.466	1.02 [0.94-1.11]	0.560
4	0.94 [0.85-1.04]	0.250	1.02 [0.94-1.10]	0.629
5	0.94 [0.85-1.05]	0.269	1.02 [0.94-1.11]	0.593
**BMI** (kg/m^2)				
Normal	Ref		Ref	
Underweight	0.69 [0.52-0.91]	0.010	0.98 [0.82-1.18]	0.841
Overweight	1.07 [0.99-1.15]	0.094	0.97 [0.92-1.03]	0.393
Obese	1.14 [1.05-1.24]	0.001	0.95 [0.90-1.01]	0.126
Unknown	0.69 [0.51-0.92]	0.011	0.92 [0.74-1.15]	0.453
**Smoking during pregnancy**				
Never	Ref		Ref	
Ex-smoker	1.05 [0.96-1.15]	0.272	1.08 [1.00-1.16]	0.049
Current smoker	0.97 [0.88-1.07]	0.498	1.17 [1.08-1.26]	<0.001
Unknown	0.61 [0.56-0.66]	<0.001	1.37 [1.29-1.46]	<0.001
**Asthma medications (year before pregnancy)**				
SABA only	Ref		NA	
ICS <4/year	0.91 [0.84-0.98]	0.009	Ref	
ICS ≥4/year	0.36 [0.31-0.42]	<0.001	0.59 [0.54-0.64]	<0.001
ICS + add-on <4/year	0.96 [0.88-1.05]	0.396	0.94 [0.88-1.00]	0.044
ICS + add-on ≥4/year	NA		0.40 [0.37-0.42]	<0.001
**Asthma exacerbations (year before pregnancy)**				
None	Ref		Ref	
≥1	1.22 [1.12-1.34]	<0.001	1.43 [1.34-1.52]	<0.001
**Asthma annual review (year before pregnancy)**				
No	Ref		Ref	
Yes	0.99 [0.92-1.06]	0.747	0.95 [0.90-1.00]	0.055

Blood eosinophil count (x10^9/L)				
<0.3	Ref		Ref	
≥0.3	1.17 [1.09-1.26]	<0.001	0.95 [0.90-1.00]	0.053
Unknown	1.13 [1.04-1.24]	0.005	0.89 [0.84-0.96]	0.001
**Atopy**				
No	Ref		Ref	
Yes	1.02 [0.96-1.09]	0.561	0.93 [0.88-0.98]	0.004
**Anxiety/Depression**				
No	Ref		Ref	
Yes	1.01 [0.94-1.08]	0.731	1.10 [1.05-1.16]	<0.001
**Multigravida**				
No	Ref		Ref	
Yes	0.99 [0.91-1.07]	0.765	0.96 [0.90-1.01]	0.132

Abbreviation: OR=odds ratio, 95%CI= 95% confidence interval, IMD= Index of Multiple Deprivation, BMI=body mass index (kg/m^2), ICS= Inhaled Corticosteroids, SABA= Short-Acting Beta-Agonists
